# Physical activity and quality of life in children with idiopathic toe walking: a cross sectional study

**DOI:** 10.1186/s12887-022-03583-w

**Published:** 2022-09-13

**Authors:** Antoni Caserta, Sarah Reedman, Prue Morgan, Cylie M. Williams

**Affiliations:** 1 Department of Physiotherapy, School of Primary and Allied Health Care, Monash University, 47 - 49 Moorooduc Hwy, Frankston, VIC 3199 Australia; 2 Monash Health Community, 140-155 Sladen Street, Cranbourne, VIC 3977 Australia; 3 Queensland Cerebral Palsy and Rehabilitation Research Centre, Child Health Research Centre, Faculty of Medicine, The University of Queensland, Brisbane, Queensland Australia; 4 School of Primary and Allied Health Care, Monash University, 47 - 49 Moorooduc Hwy, Frankston, VIC 3199 Australia

**Keywords:** Idiopathic toe walking, Gait, Physical activity, Quality of life, Paediatrics

## Abstract

**Objectives:**

To determine if children with idiopathic toe walking (ITW) reach Australian 24-hour movement guidelines. Additional objectives were to identify any factors associated with moderate to vigorous physical activity time of children with ITW.

**Design:**

Cross sectional.

**Setting:**

Private practice, public health outpatient, community clinics.

**Participants:**

Children between 4 and 14 years, who toe walked and had no medical conditions known to cause ITW.

**Outcome measures:**

Physical activity intensity, sedentary behaviour and sleep data were collected via an ActiGraph. Physical activity level intensity data were triangulated with the Child Leisure Activities Study Survey (CLASS) to highlight the subjective nature of parent-reported measures. Health related quality of life information was collected using the Parent-Proxy and Child-Self Report Pediatric Quality of Life Inventory (PedsQL) 4.0 Generic Core Scale. Regression analyses were used to explore individual factors associated with moderate to vigorous physical activity.

**Results:**

Twenty-seven participants, 17(63%) male, age mean = 6.62 (SD = 2.29) years, provided information on physical activity (CLASS *n* = 18, ActiGraph *n* = 22), physical functioning and psychosocial functioning domains on the PedsQL (Parent-Proxy *n* = 25, Child n = 22). All participants exceeded Australian recommendations for physical activity, 44% (8/18) met recommended screen time amounts, and two (9%) met recommended sleep times. The Child-Self Report PedsQL scale score of social functioning was the only factor associated with an increase in physical activity (Coef = 0.48, 95%CI = 0.09 to 0.87, *p* = 0.019).

**Conclusion:**

Participants achieved high levels of daily moderate to vigorous physical activity, and this was associated with social functioning. Given current uncertainty regarding benefits and effectiveness of treatment choices for children who have ITW, these findings should encourage clinicians to consider how their treatment recommendations interact with the PA level and sleep of children with ITW. Any treatment choice should also be implemented with consideration of how it may impact social functioning. This study had a small sample size therefore results should be cautiously interpreted and not generalised to all children with ITW.

**Supplementary Information:**

The online version contains supplementary material available at 10.1186/s12887-022-03583-w.

## Article summary

Strengths and limitations of this study

- The study investigated physical activity and quality of life in children with idiopathic toe walking.
- Participants achieved high levels of daily moderate to vigorous physical activity
- Participant outcomes were compared to Australian 24-hour movement guidelines.
- Findings should be cautiously interpreted due to sample size.
- Health related quality of life impacts were associated with physical activity, but causal relationship was not examined.

## Introduction

Idiopathic toe walking (ITW) is an exclusionary gait disorder, where a child presents with a limited or absent heel strike [1, 2]. ITW is diagnosed when all other conditions known to cause toe walking are eliminated [1, 2]. ITW is estimated to impact 5% of healthy children at 5 years of age [3], is present in both sexes [4] and commonly associated with ankle equinus [5]. Ankle equinus in any population is thought to be a major contributor to lower limb or foot pain [6], poor motor skills [7], and low participation in sports and leisure time physical activities [8]. Physical activity levels however have not yet been examined in children with ITW [9].

Physical activity is defined as ‘any bodily movement produced by skeletal muscles that results in energy expenditure’ [10]. Intensity of physical activity can vary from sedentary, through to light, moderate and vigorous intensities which are defined according to their ratio of a person’s resting energy expenditure [10]. Physical activity is important for prevention of obesity [11] and bone health [12]. A child’s physical activity is influenced by a number of modifiable (psychological, cognitive, emotional, healthy diet) and non-modifiable (gender, male parent weight, previous physical activity engagement) factors [13]. Australian guidelines provide recommendations for minimum amounts of physical activity at varying intensities for children across different age groups. These movement guidelines are for a minimum of 60 minutes of energetic play, which align with moderate to vigorous physical activity (MVPA) for children above the ages of 3 years. These guidelines also recommend several hours of light physical activity (LPA), limiting sedentary recreational screen time to less than 120 minutes per day and block sleep time designed for different age groupings [13]. Additional recommendations to maximise the impact of physical activity on health include breaking up long periods of sitting, consistent sleep hygiene and an uninterrupted sleep each night [14]. Meeting these guidelines are not only important for a child’s health but also for their quality of life and psychological wellbeing [15, 16].

Physical activity intensity and type (including sedentary behaviour) is commonly measured using accelerometery, owing to its convenience and acceptability compared to the criterion standard of indirect calorimetry (oxygen consumption). Accelerometry has been utilised to assess habitual physical activity in children who toe walk from medical conditions such as cerebral palsy and autism. Studies concluded that children who toe walk with these diagnoses were less likely to meet physical activity guidelines compared to typically developing peers [17, 18]. To our knowledge, there are no studies examining the physical activity level of children with an ITW gait using accelerometry outside of the laboratory or clinical setting.

There remains controversy as to whether ITW should be treated, or left to self-resolve [1]. Treatment decisions may be based on many factors such as impairment at the foot or ankle, any pain, or a child’s ability to participate, yet the range of these factors are not commonly reported within treatment studies about ITW [1]. Developing a greater understanding of any burden of disease associated with this condition could assist health professionals and family navigate the varied treatment options and help inform treatment timing decisions. This study aimed to explore any burden ITW has by describing if children with an ITW gait meet Australian 24-hour movement guidelines, and identifying any factors associated with moderate to vigorous physical activity time. Our hypothesis was that children with ITW would not meet the Australian guidelines.

## Objectives of this study

To determine if children with ITW reach Australian 24-hour movement guidelines. Additional objectives were to identify if there are any modifiable (body mass index, screen time, quality of life) or non-modifiable (gender, age) factors that were associated with the MVPA time of children with ITW.

## Methods

This study was cross–sectional in design, and was approved by the Monash Health Human Research Ethics Committee (MHREC:15405A). All parents/carers of participants provided written informed consent, and child participants verbally assented prior to participation. This present study was nested within a larger study exploring the strength and range of motion of foot and leg muscles of children with an ITW gait. Data collection about physical activity and quality of life was the secondary outcome of the larger study examining potential physical features associated with the gait.

### Patient and public involvement

There was no patient or public involvement in the design of this study. Parents of participants were provided with summary outcomes if requested.

### Participants

Participants were recruited from private practice clinics, public health outpatient and community clinics via advertising to clinicians. Eligible participants were between 4 and 16 years, otherwise healthy and demonstrated a consistent ITW gait pattern. The ITW exclusionary diagnosis was made through either a multidisciplinary (medical/allied health) hospital clinic or via combined assessment by two experienced health professionals (physiotherapist and podiatrist) using a validated exclusionary tool [2]. These assessments considered cognitive delay, gross motor skill and risk factors of the toe walking gait being related to a neurological, orthopaedic or developmental condition. Prior to inclusion, the clinicians also observed gait and that the child displayed consistent or intermittent toe walking but was able to get their heels to the ground on request. Participants were excluded if they had any active treatment prescribed by a health professional for their toe walking gait within the previous 12 months. Active treatment was considered as serial casting, surgery, gait aids or motor control interventions that were monitored and modified by a health professional. This was to minimise any confounding influence on physical activity.

### Outcome measures

#### Participant demographics

The age (years), gender, weight (kg) using a calibrated scale (Anko Electronics, California, USA), height (m) using a stadiometer (Seca, Hamburg, Germany) of each participant were documented at an initial appointment. The data collection period in relation to local school holidays or school term was also documented.

#### Habitual physical activity and sleep

Habitual physical activity and sleep data were collected through accelerometry using a single Actigraph worn on the non-dominant wrist (wActiSleep-BT; Actigraph LLC. Pensacola, FL 32502, USA). Participants were fitted with the Actigraph by the investigator, and shown how to tighten the band and where it should be located on their wrist at the appointment. They were instructed to wear the Actigraph from the time of appointment, until a set day. This set timepoint was remotely controlled and set and for 7 days. The child was instructed to keep the Actigraph on at all times except for bathing or water-based activities. Using the Actigraph in this way is a form of measuring physical activity and validated in a paediatric population [19]. The variables of interest were physical activity intensity (e.g. time spent in MVPA, total sleep amount, wear-time validation). A valid wear day was considered as > 10 hours of non-sleep time and observations were included when the participant wore the monitor for > 4 days [20].

Parents also completed the paper-based proxy-activity Child Leisure Activities Study Survey (CLASS) questionnaire during the data collection week to capture information about the type (mode) of participation in physical activities [21]. This proxy questionnaire has demonstrated reliability in measurement of the type, frequency, and duration of children’s physical activity [21]. Parents were instructed to document the amount (minutes) of physical activity and leisure activity participation daily on the form provided, including community-based activity, school sports, play-based activity, and leisure activities. Additional parental free text commentary on activity was captured and included where relevant.

#### Pediatric quality of life inventory (PedsQL)

Health related quality of life scores were measured with the age appropriate Parent-Proxy and Child-Self Report PedsQL4.0 Generic Core Scales [22]. These scales consist of 23 questions that encompass physical functioning (8 items), emotional functioning (5 items), social functioning (5 items) and school functioning (5 items) domains. The scale asks how much of a problem each item has been during the past 1 month, with the parent or child scoring on a 3 point or 5 point Likert scale respectively, corresponding to the age group of the scale. For the current study, the scales responding to ages 5-7 years (young child), 8-12 years (child) and 13-18 years (adolescent) scale were used as per age of the child. All questions were the same for each scale, with adjusted developmentally appropriate language and scoring. Scores were reversed and totalled so that a higher score in each domain indicates better HRQoL (Health-Related Quality of Life). The Child-Self and Parent-Proxy Report scales have reported satisfactory validity and reliability [22].

#### Procedure

Participants were recruited from private practice clinics, public health outpatient and community clinics, in Melbourne, Victoria, Australia. Potential participants were initially screened against the eligibility criteria by a clinician researcher who had > 8 years working in public health community-based paediatric gait screening clinics. Following parent and participant consent, demographic data, time period in relationship to school term were collected, and HRQoL surveys were completed. Participants were fitted with the ActiGraph on the non-dominant wrist, with a flexible fabric wristband. Participants were instructed to wear the ActiGraph for 24 hours a day for a period of 7 consecutive days (excluding showering, bathing or swimming) to maximise pre-determined valid wear-time decisions during analysis [20]. To minimise wearer bias, valid wear-time was not communicated to participants. Parents were provided with the paper-copy of the CLASS and instructed in its completion at the end of every day for 7 consecutive days.

#### Analysis

The accelerometers were initialized to sample data at 60 Hz frequency. The cut points for the vertical axis as validated by Chandler et al. (2015) were applied [23] based on the wearing position (non-dominant wrist). Chandler et al. (2015) cut points were chosen because there is currently no published consensus on the most appropriate cut points to apply to proprietary activity ‘count’ data extracted from wrist-worn accelerometers for the classification of MVPA in children. Sleep periods (and associated variables) were identified with the ActiLife version 6.13.4 (ActiGraph LLC, Pensacola, Florida) software using the Sadeh [24] algorithm and ActiGraph automated sleep period detection feature. Sleep period data were then visually inspected and then manually combined (where multiple sleep periods were detected in a single night) or deleted (where sleep periods were erroneously detected instead of daytime non-wear). Then, daytime non-wear was identified using the Troiano (2007) algorithm within ActiLife. Activity ‘count’ data was then downloaded using a 5 second epoch length and data were exported to comma separated values files and imported to Stata SE version 16.0. Finally, sleep periods and non-wear times were removed and cut points for the vertical axis were applied to each epoch to determine total time spent in MVPA. ActiGraph physical activity data was initially summarised and rated against the Australian recommended age-matched guidelines for MVPA, limiting sedentary recreational screen time to less than 120 minutes per day and uninterrupted sleep.

Participant demographic data, CLASS data and PedsQL total scale and individual scale scores were described in means (SD), frequencies (%) and ranges where appropriate. Where available, demographic data were compared to normative data either descriptively or using two sample t-tests and reporting the mean difference (MD), and 95% confidence intervals (95% CI) with published normative values [22, 25]. Univariate linear regression analyses were performed to explore any associations between the dependent variable, MVPA and independent variables known to impact moderate to vigorous physical activity. These variables included age, gender [13], BMI [26], screen time (calculated from the CLASS), and psychological, cognitive and emotional impacts [13] as determined by the PedsQL scales. All variables remaining were significant at *p*-value < 0.05.

As this was a nested study currently unpublished, the original sample size was calculated based on the larger study of active range of motion and strength measures. A sample size of 26 participants was calculated to achieve 80% power, and to detect an effect size of 0.81 as a result of differences in ankle range of motion (primary outcome) between the ITW cohort and their non-toe walking peers using an α criterion of 0.05 [27]. Data were analysed with Stata 13 (Statacorp, LP).

## Results

### Participants

Twenty-seven children participated (Table 1), ranging in age from 4 to 13 years. Eighteen parents returned the CLASS fully completed, 25 parents completed the Parent-Proxy Report PedQL, and 22 child participants completed the age appropriate Child-Self Report PedsQL. All 27 participants wore the ActiGraph, however five participants did not register sufficient valid wear time to be included for analysis, therefore 22 participants’ Actigraph data were included in the final analysis.

**Table 1:** Characteristics of 27 participants with ITWCharacteristic

	Mean (SD) or *n(%)*
Age (years)	6.62 (2.29)
Gender (male)	17 *(63%)*
BMI (Kg/m^2^) & *z-score mean (SD)*	17.12 (2.67)^a^, *0.45 (1.23)*
*Underweight*	*1 (3.8%)*^a^
*Healthy weight range*	*19 (73.1%)*^a^
*Overweight or obese*	*6 (23.1%)*^a^
Data collection period (within school term)	20 *(74.07%)*

^a^BMI calculated by Centers of Disease Control and Prevention [25]

### Accelerometry and parent report of physical activity

Table 2 describes the Australian recommendations for activity and sleep for children compared to participant results. While all study participants exceeded the Australian recommendations for physical activity, more than half (66%, *n* = 10/18) had more screen time than recommended, and only 9% (*n* = 2/22) met the total sleep time recommendations. Supplementary data file 1 provides a summary of the types of physical activity and leisure options that children within the study participated in during the data collection period.

**Table 2:** Australian 24-hour movement guidelines, and results from children with ITW

	Australian recommendations [14]	ActiGraph data Mean (SD), *Range* *Total cohort* *(n = 22)*	CLASS Mean (SD), range *Total cohort* (*n* = 18)	CLASS Mean (SD), range *School term cohort* only (*n* = 13)	CLASS Mean (SD), range *Holiday cohort only* (*n* = 5)	Met recommendations n (%)
MVPA (minutes)/ day	**Minimum of 60 minutes**	105.45 (24.53), *70.24 to 170.01*	147.02 (77.66), 12.86 to 295	123.62 (69.80), 12.86 to 197.14	195 (84.08), 91.43 to 295	22/22 (100%)
Screen time (minutes) data averaged/ day	**< 120**	NC	151.90 (98.34), *15 to 334.29*	133.52 (93.81), 15 to 333.29	199.71 (103.94), 115.71 to 317.14	8/18 (44%)
Total sleep time (minutes)/night	**4-years: 600**	445.0881 (79.87), *325.1 to 518*	NC	NC	NC	0/6 (0%)
	**5–13 years: 540**	464.157 (64.52), *350.43 to 578.29*	NC	NC	NC	2/16 (13%)

*NC* Not collected using specified outcome

The parent reports of participation in physical activities were different in frequency between school terms and holidays, with all children exceeding the Australian physical activity recommendations in both time periods. Unsolicited additional qualitative comments from parents provided on the CLASS included *“does not stop moving”,* and *“don’t walk at all in their day but run[s] everywhere”.*

### Child and parent proxy reported quality of life

There were 25 parents who completed the Parent-Proxy Report PedsQL and 22 participants completed the age appropriate Child-Self Report PedsQL. No participants or parents missed any individual question therefore conventional scoring was applied. Table 3 provides the summary of the PedsQL total scale score and functioning scales compared to published normative data.

**Table 3:** PedsQL participant data with comparisons to published normative values [22]

	Parent Proxy Report *N* = 25 Mean (SD), Range	Normative data^a^ *N* = 8713 Mean (SD)	Mean Difference, (95% CI), p	Child Self Report *N* = 22 Mean (SD) Range	Normative data^a^ *N* = 5079 Mean (SD)	Mean Difference, (95% CI), p
Total score	73.70 (13.60) 47.83 to 95.65	82.29 (15.55)	8.59, (2.49 to 14.69), 0.006	61.17 (19.13) 28.26 to 97.83	83.91 (12.47)	22.74, (17.50 to 27.98), < 0.001
Physical Functioning	78.88 (15.2) 50 to 100	84.08 (19.70)	5.20, (−2.53 to 12.93), 0.187	66.19 (22.46) 6.25 to 93.75	87.77 (13.12)	21.58, (16.06 to 27.10), < 0.001
Emotional Functioning	61.60 (16.24) 45 to 95	81.20 (16.40)	19.60, (13.16 to 26.04), < 0.001	50.00 (24.49) 10 to 100	79.21 (18.02)	29.21, (21.65 to 36.77), < 0.001
Social Functioning	79.00 (18.08) 45 to 100	83.05 (19.66)	4.05, (−3.67 to 11.77), 0.304	60.91 (23.71) 20 to 100	84.97 (16.71)	24.06, (17.03 to 31.09), < 0.001
School Functioning	72.2 (16.83) 35 to 100	78.27 (19.64)	6.07, (−1.63 to 13.78), 0.122	64.55 (25.13) 20 to 100	81.31 (16.09)	3.45, (10.00 to 23.52), < 0.001

^a^ Healthy population data [22]

### Impact of physical activity on quality of life

Univariate output is provided in Table 4. Only the Child-Self Report score for the social functioning score was associated with MVPA. This analysis determined for every increase of 1 minute of MVPA, there was an increase of 0.48 points in the social functioning scale (Coef = 0.48, 95%CI = 0.09 to 0.87, *p* = 0.019).

**Table 4:** Univariate output for VM MVPA

Modifiable and non-modifiable model variables	MVPA Coef, [95% CI], p
Weight (Kg)	−0.30, [− 1.65 to 1.05], 0.650
BMI (Kg/m^2)^	0.51, [− 3.49 to 4.51], 0.793
Age (years)	− 0.80, [− 5.46 to 3.85], 0.723
Screen time (minutes)	0.10, [− 0.06 to 0.25], 0.195
Physical functioning (Parent-Proxy)	0.29, [− 0.45 to 1.04], 0.412
Emotional functioning (Parent-Proxy)	0.44, [−0.32 to 1.21], 0.239
Social functioning (Parent-Proxy)	0.19, [−0.46 to 0.84], 0.560
School functioning (Parent-Proxy)	0.52, [−0.09 to 1.13], 0.092
Physical functioning (Child-Self)	0.45, [0.00 to 0.89], 0.050
Emotional functioning (Child-Self)	0.40, [−0.02 to 0.83], 0.063
Social functioning (Child-Self)	0.48, [0.09 to 0.87], 0.019
School functioning (Child-Self)	0.15, [0.46 to 0.57], 0.468

## Discussion

This study investigated the physical activity of children with ITW gait against the Australian 24-hour movement guidelines. Participants within this study achieved sufficient physical activity intensity [14]. This contrasted with our hypothesis, given many children with gait disorders are less physically active than their peers, and do not meet physical activity recommendations [28, 29]. Finding physical activity levels in this cohort higher than the minimum recommended levels by the guidelines may provide some insights into the less favourable outcomes of management of children with ITW reported by clinicians [6] and parents [30]. Higher levels of physical activity in children with ITW may prompt dosage consideration of any prescribed treatment. For example, children with ITW may need to practice a prescribed new movement more frequently than other children who move less in order to establish new movement patterns. This may be particularly pertinent in older children with ITW who may have more ingrained movement patterns.

Although the children within this study all met the physical activity guidelines for MVPA, they generally spent greater than the recommended time in front of screens, and the majority did not meet the sleep guidelines. Increased screen time [31] and reduced sleep [31] are risk factors for obesity within children, although prevalence of high BMI was not greater than in the general population for children in this cohort. To our knowledge, sleep variables have not been collected with ITW cohorts before, therefore comparison of this data to similar populations was not possible. Other children who toe walk with diagnoses such as autism, regularly report difficulties with sleep onset, insomnia and sleep disruption [32]. Further research may consider investigating these sleep variables in children with ITW. It is commonly accepted that some children with ITW display different sensory behaviours to children without toe walking gait, and altered sleep patterns as well as reduced sleep may exist [7]. Sleep quality and quantity of children with ITW, particularly where uncomfortable interventions such as serial casting may impact sleep hygiene should be considered as part of treatment planning.

Movement and sensory behaviours have been examined in other studies including children with ITW [7, 33]. These studies report differing behaviours thought to be related to sensory processing challenges and delays in balance and motor skill acquisition in children with ITW compared to typically developing children [7, 33]. The differing behaviours included exhibiting greater sensory seeking behaviours or increased movement in play, such as falling, spinning or fidgeting. In this study children with ITW recorded increased movement through accelerometry, and unsolicited parent responses indicated high levels of movement all of the time, in alignment with the increase in movement reported in prior studies. In the future, researchers may consider collecting parent or teacher reported sensory processing information to understand any relationship with the elevated physical activity observed in children with ITW gait.

Psychological, cognitive, and emotional barriers are known to influence physical activity in children [13]. The present results suggest that the ability of children with ITW to participate with their peers may be associated with their level of moderate to vigorous physical activity. This relationship is similar to that seen between physical activity and social functioning in children with cerebral palsy [34]. The design of the present study does not permit the establishment of a causal link between physical activity enhancing perceived quality of life related to social functioning. Nevertheless, this association is one that researchers and clinicians should consider when designing and delivering interventions to impact either variable. Clinicians may consider asking children about play, friendships, and how they interact with their peers during play, to understand the impact of ITW gait on quality of life outcomes.

Participants with ITW in this study reported lower quality of life (total PedsQL scale score) within all individual functional domains when compared to healthy peers [22]. In comparison, parents of children with ITW reported their children to have lower quality of life total score and emotional domain lower score only when compared to healthy peers [22]. In prior research, children with cerebral palsy scored themselves as having lower physical functioning, but higher scores in all psychosocial domains compared to children with ITW in this study [35]. In comparison, parents of children with cerebral palsy indicated their child had greater problems in most domains of the PedsQL compared to parents of children with ITW [35]. Similar to children with ITW in this study, children with cerebral palsy and their parents have discordant HRQoL reports using the PedsQL, endorsing PedsQL instructions that encourage both parent proxy and child self-report to be collected. Parent-Proxy Report PedsQL scale scores of children with autism who may also toe walk were also similar to the Parent-Proxy Report scale scores of children with ITW in this study in all domains except social functioning. Whereas parents of children with autism rated their child as having lower quality of life scores than those with ITW [36]. Whilst not statistically analysed, the scores taken from Child-Self Report reports of children with ITW in this study appear similar to those reported by children with a range of chronic health conditions [22]. Clinicians should consider using a similar HRQoL lens when providing care for children with ITW gait as they would with children with cerebral palsy, autism, or chronic health conditions when determining treatment decisions. Future research should also consider determining the minimal clinical important difference.

There are several limitations in this study. Non-dominant wrist worn accelerometer is generally favoured in children’s physical activity studies to increase validity of activity intensity [37], valid wear-time and adherence [38]. However, it is unknown if this data collection method may overestimate physical activity. Therefore, results presented should be interpreted with caution until replicated in a study with cut points specifically validated in children with ITW gait. A further limitation is the small sample size, as it was calculated based on power estimates relating to the larger study based on a different outcome measure. This, combined with the missing data resulted in minimising statistical analysis and comparisons to normative populations so not to overemphasise or generalise results to the entire ITW population. Future studies with larger sample sizes should enable in-depth analysis of any association between factors known to impact physical activity levels.

## Conclusion

ITW is thought to be a benign and transient gait pattern requiring minimal intervention from health professionals. Although some children may reduce or cease toe walking as they get older, for those that continue, the high level of MVPA undertaken may further ingrain this gait pattern. If high levels of daily physical activity are present, this may impact the efficacy of exercise dosage or therapy type on influencing impairment or functional outcomes. The impact of persistent toe walking on quality of life may also need to be considered when approaching a decision to treat.

## Supplementary Information

**Additional file 1: Supplementary data table 1.** Frequency of types of the types and number of children participating in the CLASS categories of physical activity and screen time categories.

## Data Availability

Request for further details of the data set and queries relating to data sharing arrangements may be submitted to Antoni Caserta (antoni.caserta@monash.edu). Aggregate or summarised data may be shared based on reasonable request.

## References

[1] Caserta AJ, Pacey V, Fahey MC, et al. Interventions for idiopathic toe walking. Cochrane Database Syst Rev. 2019;(10). 10.1002/14651858.CD012363.pub2 PMC6778693 31587271

[2] Williams CM, Tinley P, Curtin M (2010). The toe walking tool: a novel method for assessing idiopathic toe walking children. Gait Posture.

[3] Engstrom P, Tedroff K (2012). The prevalence and course of idiopathic toe-walking in 5-year-old children. Pediatrics.

[4] Engelbert R, Gorter JW, Uiterwaal C (2011). Idiopathic toe-walking in children, adolescents and young adults: a matter of local or generalised stiffness?. BMC Musculoskelet Disord.

[5] Williams C, Tinley PD, Curtin M (2013). Foot and ankle characteristics of children with an idiopathic toe-walking gait. J Am Podiatr Med Assoc.

[6] Williams CM, Gray K, Davies N (2020). Exploring health professionals' understanding of evidence-based treatment for idiopathic toe walking. Child Care Health Dev.

[7] Williams CM, Tinley P, Curtin M (2014). Is idiopathic toe walking really idiopathic? The motor skills and sensory processing abilities associated with idiopathic toe walking gait. J Child Neurol.

[8] Akkurt L, Gürbüz İA, Karaduman A (2019). Lower limb flexibility in children with Duchenne muscular dystrophy: effects on functional performance. Pediatr Exerc Sci.

[9] Caserta A, Morgan P, Williams C (2019). Identifying methods for quantifying lower limb changes in children with idiopathic toe walking: a systematic review. Gait Posture.

[10] Caspersen CJ, Powell KE, Christenson GM. Physical activity, exercise, and physical fitness: definitions and distinctions for health-related research. Public Health Rep. 1985;100(126). PMC1424733 3920711

[11] Park E, Kwon M (2018). Health-related internet use by children and adolescents: systematic review. J Med Internet Res.

[12] Boreham CA, McKay HA (2011). Physical activity in childhood and bone health. Br J Sports Med.

[13] Sallis J, Prochaska J, WT (2000). A review of correlates of physical activity of children and adolescents. Med Sci Sports Exerc.

[14] Okely AD, GD, Hesketh KD, Santos R, Loughran SP, Cliff DP. A collaborative approach to adopting/adapting guidelines. The Australian 24-Hour Movement Guidelines for the Early Years (Birth to 5 years): An Integration of Physical Activity, Sedentary Behaviour, and Sleep. BMC Public Health. 2017;17(5):167–90. 10.1186/s12889-017-4867-6 PMC5773882 29219094

[15] Müller-Riemenschneider F, RT, Nocon M, Willich SN (2008). Long-term effectiveness of interventions promoting physical activity: a systematic review. Prev Med (Baltim).

[16] Wafa SW, Shahril MR, Ahmad AB (2016). Association between physical activity and health-related quality of life in children: a cross-sectional study. Health Qual Life Outcomes.

[17] Bratteby Tollerz LU, FA, Olsson RM, Lidstrom H, Holmback U (2015). Children with cerebral palsy do not achieve healthy physical activity levels. Acta Paediatr.

[18] MacDonald M, LC, Ulrich DA (2013). The relationship of motor skills and social communicative skills in school-aged children with autism spectrum disorder. Adapted physical activity quarterly. 2013 Jul 1. The relationship of motor skills and social communicative skills in school-aged children with autism spectrum disorder. Adapt Phys Act Q.

[19] van Loo CM, OA, Batterham MJ, Hinkley T, Ekelund U, Brage S, Reilly JJ, Trost SG, Jones RA, Janssen X, Cliff DP (2018). Wrist acceleration cut-points for moderate-to-vigorous physical activity in youth. Med Sci Sports Exerc.

[20] Migueles JH, C-SC, Ekelund U, Nyström CD, Mora-Gonzalez J, Löf M, Labayen I, Ruiz JR, Ortega FB (2017). Accelerometer data collection and processing criteria to assess physical activity and other outcomes: a systematic review and practical considerations. Sports Med.

[21] Telford A, SJ, Jolley D, Crawford D (2004). Reliability and validity of physical activity questionnaires for children: the Children’s leisure activities study survey (CLASS). Pediatr Exerc Sci.

[22] Varni JW, BT, Seid M, Skarr D (2003). The PedsQL™* 4.0 as a pediatric population health measure: feasibility, reliability, and validity. Ambul Pediatr.

[23] Chandler JL, Brazendale K, Beets MW, et al. Classification of physical activity intensities using a wrist-worn accelerometer in 8–12-year-old children. Pediatric Obesity. 2016;11(2):120–7. 10.1111/ijpo.12033 25893950

[24] van Kooten JA, JS, Heymans MW, de Vries R, Kaspers GJ, van Litsenburg RR. A meta-analysis of accelerometer sleep outcomes in healthy children based on the Sadeh algorithm: the influence of child and device characteristics. Sleep Med Rev. 2021;44(4):zsaa231. 10.1093/sleep/zsaa231 33161428

[25] Center of Disease Control and Prevention. CDC growth charts Center of Disease Control and Prevention: Center of Disease Control and Prevention; 2021. Available from: https://www.cdc.gov/growthcharts/cdc_charts.htm. Accessed 23/06 2021.

[26] Metcalf B, Henley W, Wilkin T. Effectiveness of intervention on physical activity of children: systematic review and meta-analysis of controlled trials with objectively measured outcomes. (EarlyBird 54), Bmj. 2012;345. 10.1136/bmj.e5888 23044984

[27] Williams C, Tinley P, Curtin M (2012). Foot and ankle characteristics of children with an idiopathic toe walking gait. J Foot Ankle Res.

[28] Carlon SL, Taylor NF, Dodd KJ, et al. Differences in habitual physical activity levels of young people with cerebral palsy and their typically developing peers: a systematic review. Disabil Rehabil. 2013;35(8):647–55. 10.3109/09638288.2012.715721 23072296

[29] Thomas S, Hinkley T, Barnett L. Young children with ASD participate in the same level of physical activity as children without ASD: implications for early intervention to maintain good health. J Autism Dev Disord. 2019;49(8):3278–89. 10.1007/s10803-019-04026-9 31079278

[30] Williams C, Robson K, Pacey V (2020). American and Australian family experiences while receiving a diagnosis or having treatment for idiopathic toe walking: a qualitative study. BMJ Open.

[31] Magee C, CP, Iverson D (2014). Lack of sleep could increase obesity in children and too much television could be partly to blame. Acta Paediatr.

[32] Souders MCZS, Eriksen W, Sinko R, Connell J, Kerns C, Schaaf R, Pinto-Martin J (2017). Sleep in children with autism spectrum disorder2017. Curr Psychiatry Rep.

[33] Chu V, AL. Sensory-processing differences in children with idiopathic toe walking (ITW). Am J Occup Ther. 2020;1(74), (4_Supplement_1):7411505130p1. 10.1177/19437676261464097 PMC13640398 42698309

[34] Maher CA, TM, Ferguson M (2016). Physical activity predicts quality of life and happiness in children and adolescents with cerebral palsy. Disabil Rehabil.

[35] Varni JW, BT, Berrin SJ, Sherman SA, Artavia K, Malcarne VL, Chambers HG (2006). The PedsQL in pediatric cerebral palsy: reliability, validity, and sensitivity of the generic Core scales and cerebral palsy module. Dev Med Child Neurol.

[36] Kuhlthau K, OF, Hall TA, Sikora D, Kovacs EA, Delahaye J, Clemons TE (2010). Health-related quality of life in children with autism spectrum disorders: results from the autism treatment network. J Autism Dev Disord.

[37] Johansson E, LL, Marcus C, Hagströmer M (2016). Calibration and validation of a wrist-and hip-worn actigraph accelerometer in 4-year-old children. PLoS One.

[38] Rowlands AV, Rennie K, Kozarski R (2014). Children's physical activity assessed with wrist-and hip-worn accelerometers. Med Sci Sports Exerc.

