# Supplementary material for: Physical activity and quality of life in children with idiopathic toe walking: a cross sectional study
Source: BMC Pediatr. 2022 Sep 13;22:544. doi: 10.1186/s12887-022-03583-w (PMC9472422; doi:10.1186/s12887-022-03583-w)
Supplement: Supplementary file 1 — Additional file 1: Supplementary data table 1. Frequency of types of the types and number of children participating in the CLASS categories of physical activity and screen time categories. [file 12887_2022_3583_MOESM1_ESM.docx]

Supplementary data table 1. Frequency of types of the types and number of children participating in the CLASS categories of physical activity and screen time categories

|  | Number of occasions  Monday-Friday | Number of children participating in activity  Monday - Friday | Number of occasions Saturday-Sunday | Number of children participating in activity  Saturday - Sunday |
| --- | --- | --- | --- | --- |
| Aerobics | 7 | 2 | 1 | 1 |
| Dance | 20 | 7 | 7 | 4 |
| Calisthenics/ gymnastics | 12 | 6 | 5 | 4 |
| Tennis/bat tennis | 2 | 1 | 2 | 2 |
| Australian Football (AFL) | 2 | 1 | 2 | 2 |
| Soccer | 13 | 4 | 4 | 3 |
| Basketball | 9 | 3 | 3 | 2 |
| Cricket | 3 | 2 | 2 | 2 |
| Netball | 3 | 2 | 3 | 2 |
| Swimming laps | 2 | 2 | 2 | 2 |
| Swimming for fun | 6 | 5 | 6 | 6 |
| Down ball/4 square | 5 | 2 | 2 | 1 |
| Tag/chasey | 31 | 9 | 6 | 4 |
| Skipping rope | 2 | 1 | 1 | 1 |
| Scooter | 36 | 10 | 16 | 9 |
| Skateboarding | 3 | 1 | 3 | 2 |
| Bike riding | 26 | 10 | 19 | 11 |
| Household chores | 31 | 7 | 14 | 7 |
| Play on playground equipment | 52 | 15 | 13 | 10 |
| Play in the cubby house | 17 | 7 | 9 | 6 |
| Bounce on the trampoline | 12 | 5 | 9 | 7 |
| Play with pets | 43 | 10 | 17 | 9 |
| Walk the dog | 3 | 2 | 5 | 2 |
| Walk for exercise | 13 | 5 | 6 | 5 |
| Jogging or running | 21 | 7 | 8 | 5 |
| Physical education class at school | 4 | 3 | - | - |
| Sport class at school | 17 | 9 | - | - |
| Other* | 13 | 5 | 6 | 4 |
| Watching TV / videos / DVDs | NC | 17 | NC | 17 |
| Play station / nintendo / computer games | NC | 6 | NC | 6 |
| Computer / internet | NC | 9 | NC | 9 |

*Other included fishing, karate, general play at home, gardening and participating in athletic carnival

NC – Not collected as the CLASS only asks parents to estimate the time spent in screen based activities and not the number of occasions.
